# The efficacy and safety of Chinese traditional medicine injections on patients with coronavirus disease 2019: A protocol for systematic review and meta analysis

**DOI:** 10.1097/MD.0000000000021024

**Published:** 2020-07-31

**Authors:** Yulin Li, Haonan Xu, Hui Lang, Jing Li, Lin Bi, Yanqing Li, Liang Dong, Lin Zhang, Xin Liang, Hongqiu Zhu

**Affiliations:** a Department of Andrology, The Reproductive and Women-Children Hospital, Chengdu University of Traditional Chinese Medicine, Sichuan; b Department of Evidence-Based Medicine and Clinical Epidemiology, West China Hospital, Sichuan University; c Hospital of Chengdu University of Traditional Chinese Medicine; d Chengdu Fifth People's Hospital, Chengdu, Sichuan, China.

**Keywords:** Novel coronavirus 2019, Chinese traditional medicine injections, coronavirus disease 2019, severe acute respiratory syndrome coronavirus 2

## Abstract

**Introduction:**

The pandemic caused by the coronavirus disease 2019 (COVID-19) infection has exposed vulnerable populations to an unprecedented global health crisis. Research reported that Chinese traditional medicine injections were used in patients with COVID-19 infection and showed significant effects, and there have been no systematic review and meta-analyses to investigate the effects and safety of Chinese traditional medicine injections.

**Materials and methods:**

This systematic review and meta-analysis protocol is based on the Preferred Reporting Items for Systematic Review and Meta-Analysis Protocols 2015 statement. The literature search will involve Cochran library, Web of science, PubMed, MEDLINE, Embase, China Biology Medicine Database, China National Knowledge Infrastructure Database, VIP, Wang Fang database, and China Clinical Trial Registration Center for articles and research published form December 2019. This search will include randomized controlled trials and nonrandomized studies. The Cochrane Collaboration's tool for randomized controlled trial studies and the Quality Assessment Tool for Quantitative Studies for nonrandomized studies will be used to assess the risk of bias among the studies included in the systematic review. Review Manager 5.3 software will be used for the meta-analysis, and odds ratio are calculated as the primary outcomes. Subgroup analyses will then be performed based on the characteristics of the interventions and populations included in the studies examined.

**Ethics and dissemination:**

This systematic review protocol is designed to provide evidence regarding the effects and safety of Chinese traditional medicine injections on patients with COVID-19, such evidence may be useful and important for clinical treatment decisions. The results should be disseminated through publication in a peer-reviewed journal. Since the data and results used in the systematic review will be extracted exclusively from published studies, approval from an ethics committee will not be required.

## 1 Introduction

Novel coronavirus (2019-nCoV) is raging all around the world, resulting in the death toll surging to 139,515 and confirmed cases spiking to 2,078,605, as of 2:00 am CEST, April 17, 2020, reported to WHO.^[1]^

Coronavirus disease 2019 (COVID-19) belongs to the coronavirus of genus β; however, its infection mechanism is not yet completely clear. On account of the long incubation period and highly infectivity of COVID-19 novel coronavirus poses great challenges to the prevention and treatment of diseases, while there exist no special medicine proved to be effective.^[2,3]^

Chinese traditional medicine injections are recommended by the Chinese Clinical Guidance of COVID-19 Pneumonia Diagnosis and Treatment (7th edition) published by China National Health Commission on March 4, 2020, which is including Xiyanping injection, Xuebijing injection, Reduning injection, Tanreqing injection, Xingnaojing injection, Shenfu injection, Shengmai injection, and Shenmai injection for critical ill patients.^[4]^

Chinese traditional medicine is widely used in clinical treatment. Reports show that Chinese traditional medicine injections have a certain effect in new COVID-19. The early use of traditional Chinese medicine injections can significantly shorten the course of the disease, alleviating the clinical symptoms of patients, and reducing the conversion of ordinary type to heavy and critical.^[5–7]^

Although the guideline^[4]^ mentions these traditional Chinese medicine injections can be used to treat severe patients with COVID-19, and some clinical research reported the efficiency, there is no systematic review and meta-analyses to investigate the effects and safety of Chinese traditional medicine injections. As far as the current registration situation is concerned, many clinical randomized controlled trial (RCT) trials are about to be carried out. Considering this, the present study aims to completely review the effects and safety, which will be of great significance to the clinical treatment of COVID-19.

## 2 Review objectives

The study aims to investigate whether it is effective and safe to apply Chinese traditional medicine injections on patients with COVID-19, including effective rate, all-cause mortality clinical recovery time negative time of novel coronavirus nucleic acid, etc. The results will provide evidence-based evidence for clinical treatment decisions.

## 3 Materials and methods

This is a systematic review and meta-analysis protocol, which is based on the Preferred Reporting Items for Systematic Review and Meta-Analysis Protocols (PRISMA-P) 2015 statement^[8]^ and Cochrane Collaboration Handbook.^[9]^ The data and results used in this paper are form online databases.

### 3.1 Included and excluded criteria

1. Study design: RCTs and nonrandomized studies will be included in this review.
2. Participants: Patients diagnosed with coronavirus disease 2019 (COVID-19) infection, regardless of age and gender.

Those who meet one of the following conditions can be regarded as critical patients.

Adults meet any of the following criteria:

1. Shortness of breath, risk ratio (RR) ≥ 30 times/min
2. Oxygen saturation ≥ W93% at rest
3. Alveolar oxygen partial pressure/fraction of inspiration O_2_ (PaO_2_/FiO_2_)/≤300 mm Hg (1 mm Hg = 0.133 kPa)
4. At high altitude (above 1000 m), PaO_2_/FiO_2_ should be corrected according to the following formula: PaO_2_/FiO_2_ × (atmospheric pressure [mm Hg]/760)
5. Patients whose pulmonary imaging showed significantly progression of lesion >50% within 24 to 48 hours should be treated as severe type

Children meet any of the following criteria:

1. Shortness of breath (<2 months of age, RR ≥ 60 beats/min; 2–12 months of age, RR ≥ 50 beats/min; 1–5 years old, RR ≥ 40 beats/min; >5 years old, RR ≥ 30 beats/min), excluding the effects of fever and crying
2. In the resting state, the oxygen saturation is ≤92%
3. Assisted breathing (groaning, wing flaps, tri-retraction sign), cyanosis, intermittent apnea
4. Lethargy and convulsions
5. Refusing to feed, and have signs of dehydration

Critically severe patients will require any of the following conditions:

1. Respiratory failure requiring mechanical ventilation
2. Shock
3. Patients combined with other organ failure needed intensive care unit monitoring and treatment

### 3.2 Methods

1. Interventions: Chinese traditional medicine injections reported in the guideline,^[4]^ including Xiyanping injection, Xuebijing injection, Reduning injection, Tanreqing injection, Xingnaojing injection, Shenfu injection, Shengmai injection and Shenmai injection.
2. Controls: Include no treatment, placebo, or other active treatment recommended by the guideline.
3. Outcomes: We provide the following results to reflect the efficacy and safety of traditional Chinese medicine injection.
4. Main results: effective rate and all-cause mortality.
5. Secondary results: clinical recovery time, negative time of novel coronavirus nucleic acid, mechanical ventilation time, intensive care unit length of stay, dose response of injection and adverse event rate.

### 3.3 Data source

Online databases that will be searched are as follows: Cochran library, Web of Science, PubMed, MEDLINE, Embase, China Biology Medicine Database, China National Knowledge Infrastructure Database, VIP, and Wang Fang database. The literature will be search from December 2019 with language restriction in English and Chinese. The related reference will be retrieved as well. In addition, Chinese Clinical Trial Registry Centre will also be searched.

Search term and relative variants include new coronavirus, novel coronavirus, novel coronavirus pneumonia, COVID-19, 2019-nCoV, severe acute respiratory syndrome coronavirus 2 (SARS-CoV-2), Chinese traditional medicine injection, Xiyanping injection, Xuebijing injection, Reduning injection, Tanreqing injection, Xingnaojing injection, Shenfu injection, Shengmai injection, and Shenmai injection.

Additional search strategy information is shown in Tables 1–3.

**Table 1:**
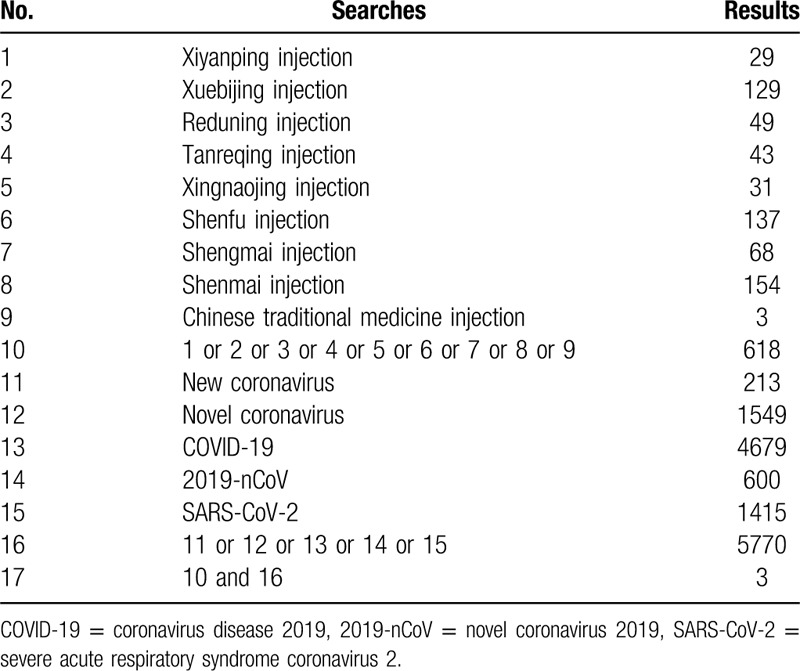
Search strategy for the Medline database.

**Table 2:**
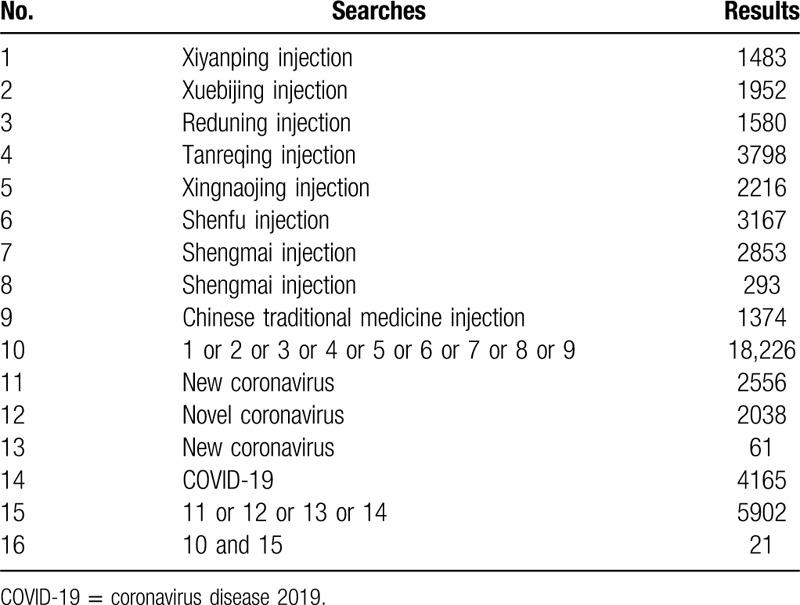
Search strategy for the China Biology Medicine Database database.

**Table 3:**
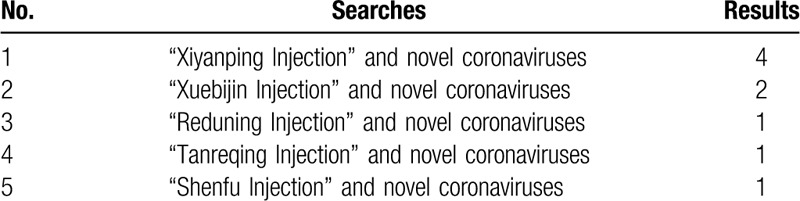
Search strategy for Chinese Clinical Trial Registry Centre.

### 3.4 Selection of studies and data extraction

**Figure 1:**
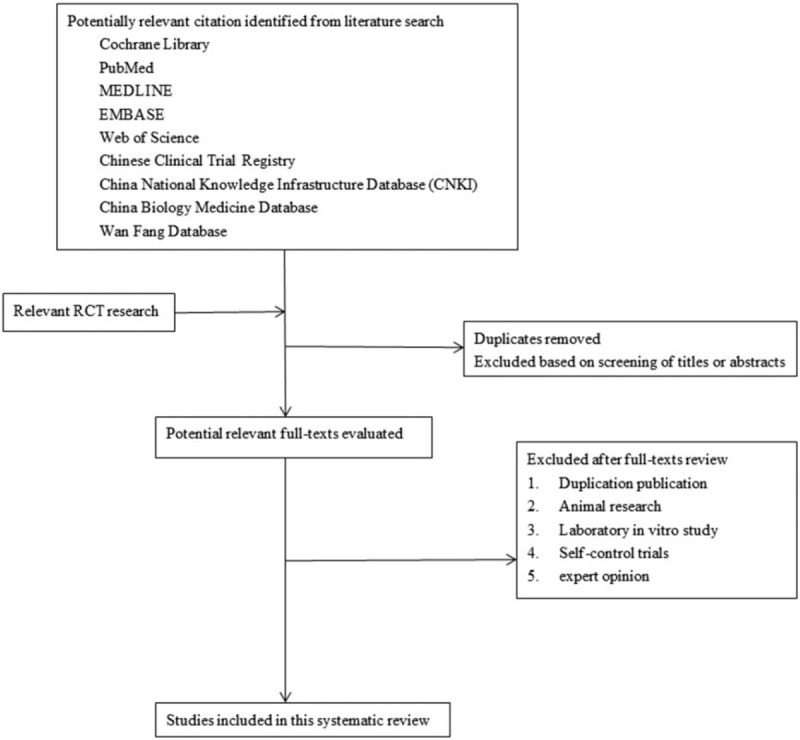
Preferred Reporting Items for Systematic Review and Meta-Analysis flow diagram for identifying, screening and determining the eligibility of and whether to include studies. RCT = randomized controlled trial.

The process of identification, selection, and the inclusion/exclusion of articles will follow the PRISMA flowchart (shown in Fig. 1).

First, Endnote X9 software will be used to filter duplicate studies. After the deduplication study, the 2 commentators will filter the literature again by reading the title, abstract and other information of the article according to the inclusion and exclusion criteria.

According to the results obtained, the full text of the target article is obtained through the database, and then 2 reviewers read the full text of the research that meets the screening criteria, reread the full text, and conduct the screening again. In case of repeated literature results, the results with the largest sample size or the longest follow-up time should be selected. When reviewers are unable to confirm the repeat study, they will contact the author for judgment. Then, the review team generated a unified data extraction table (Excel spreadsheet).

The extracted content includes the following information: research characteristics: research year, publication time, country, research background, publication type, research design, research method, and research population. Patient baseline characteristics, intervention information: dose and treatment plan, duration, route of administration; assessment results; study measurement details; bias risk assessment, and other information. The reviewers will then conduct more detailed screening and data extraction of the study. In the process of data extraction, all disagreements of reviewers will be solved through discussion with the 3rd reviewer.

After extracting data and information, professional research design tools will be used to assess research quality and bias risk. Two reviewers will use the double-blind method to independently assess and check the bias risk. If there are differences between the reviewers, they will be resolved through discussion. At the same time, they will discuss with the third party reviewer to solve the bias risk difference.

### 3.5 Risk of bias assessment

Two researchers will use the methods and cross checks recommended by Cochrane Collaboration Handbook to independently assess the bias risk of each study^[10]^: random sequence generation, task hiding, blind method, incomplete result data, selective result reporting, and other biases. In these 6 areas, we make “Yes (low bias),” “no (high bias),” and “unclear (lack of information or bias uncertainty)” judgments to assess the risk of bias in each study.

The Quality Assessment Tool for Quantitative Studies^[11]^ will be used to assess the quality of pre–post studies and non-RCTs. This tool evaluates 7 domains: selection bias, study design, confounders, blinding, data collection method, withdrawals, and dropouts.

Commentators will summarize the risks of biased judgment in each of the areas listed in different studies. Reviewers will review against the Cochrane Collaboration tool to assess the risk of bias.

### 3.6 Data analysis and synthesis

Researchers will summaries the main characteristics of each included study, including the general profile of the study, the methods, the characteristics of participants, interventions, controls, and the outcomes. A meta-analysis will be performed on studies that showed effective rate, mortality rate, or other outcomes.

For studies for which a meta-analysis is appropriate, Review Manager 5.3 software will be used to combine the odds ratio or RR with 95% confidence intervals. A fixed effects model will be used if there is no evidence of heterogeneity and a random-effects model will be used if there is significant of heterogeneity. Study heterogeneity will be assessed using the *I*^2^ statistic. Specifically, *I*^2^ values will be stratified as follows: might not be important (0–40%), may represent moderate heterogeneity (30–60%), may represent substantial heterogeneity (50–90%), and considerable heterogeneity (75–100%), the corresponding p values will also be taken into account.^[8]^

### 3.7 Subgroup analysis and meta-regression

At the time of data collection, if subgroup analysis can be performed, the reviewer will analyze according to the age and gender of the patient, intervention (different injections, different doses), controls and whether the patient has other chronic diseases. If the interaction test between related subgroups is <0.05, the reviewer will analyze and adjust it to eliminate deviation and risk, so as to ensure the stability of evidence.

### 3.8 Sensitivity analysis

Sensitivity analysis will examine the reliability and heterogeneity of the results of the mate analysis and examine the bias of the samples. The study of eliminating the risk of high bias and reducing bias can ensure the stability of the analysis results.

### 3.9 Publication bias

Following the method proposed by Sterne et al,^[12]^ funnel chart was used to measure publication bias, and Review Manager 5.3 software was used to measure publication bias. If no bias is published, the results show a symmetrical funnel-shaped reversal shape.

### 3.10 Ethics and dissemination

This systematic review protocol is designed to provide evidence regarding the effects and safety of Chinese traditional medicine injections on patients with COVID-19, such evidence may be useful and important for clinical treatment decisions. The results should be disseminated through publication in a peer-reviewed journal. Since the data and results used in the systematic review will be extracted exclusively from published studies, approval from an ethics committee will not be required.

## 4 Discussion

In Chinese traditional medicine aspect, COVID-19 is expressed as the inclusion of cold and heat, complex of dryness and wetness, and the pathologic nature of both reality and deficiency. The pathogenesis is characterized by “poison, dryness, dampness, cold, deficiency, stasis,” and inflammation as well as respiratory distress.^[13]^

Chinese traditional medicine injections were firstly recommended by the Chinese Clinical Guidance of COVID-19 Pneumonia Diagnosis and Treatment (4th edition) published by China National Health Commission on February 4, 2020, which suggested to apply Xiyanping injection in the mid-term clinical treatment, while Shenfu injection and Shengmai injection in the critical stage. Xuebijing injection can apply in both of the period.^[14]^

In the Chinese Clinical Guidance of COVID-19 Pneumonia Diagnosis and Treatment (7th edition), there are 8 injections totally, including Xiyanping injection, Xuebijing injection, Reduning injection, Tanreqing injection, Xingnaojing injection, Shenfu injection, Shengmai injection, and Shenmai injection for severe and critical patients with COVID-19. And it also recommended to combine 0.9% sodium chloride injection 250 ml with Xiyanping injection 100 mg bid, or Reduning injection 20 mL, or Tanreqing injection 40 mL bid, to patients with viral infection or mild bacterial infection.

Novel coronavirus and SARS-CoV are both belong to coronavirus of genus β. It is reported that 2019-nCoV is about 79% genetically similar to SARS-CoV.^[15]^ Researches have demonstrated that Chinese traditional medicine injections were applied in patients with SARS-CoV infection, which played a great role in ameliorating the clinical symptoms, improving the body's immunity, reducing complications, and shortening the course of disease.^[16–19]^

Thirty-four patients with COVID-19 who were treated by the combination of traditional Chinese and Western medicine in Hubei Province were treated with traditional Chinese medicine on the basis of Western medicine, including Xuebijing injection, Tanreqing injection, Shengmai injection, and Shen annotation injection. The results of clinical treatment show that the use of traditional Chinese medicine decoction and injection before the patient's admission or lung injury is more helpful to the overall recovery of patients.^[5]^ Treatment of COVID-19 with Xuebijing in Dongfeng Hospital Affiliated to Hubei Medical College, the results of retrospective analysis on the clinical efficacy of Xuebijing showed that on the basis of routine antiviral treatment, the combination of Xuebijing injection can promote the absorption of pulmonary lesions and improve the efficacy, reduce the incidence of severe cases, which may be related to the improvement of microcirculation and the effective reduction of mortality of septic shock.^[20]^ However, it is not obvious in improving the inflammation index and promoting the negative transformation of nucleic acid, and the reason needs further research.^[21]^

From 2013 to 2016, the “Centralized Monitoring Research on Clinical Safety of Xuebijing Injection” was carried out in 93 second-class (including) hospitals nationwide.^[22]^ In this study, 96 cases of adverse reactions of Xuebijing injection were detected, and the incidence of adverse reactions was 0.30%, which belonged to the level of occasional study. The results showed that Xuebijing injection had a high safety, a slight degree of adverse reactions and a good outcome under the condition of clinical rational use.^[23]^

Although the guideline mentions these traditional Chinese medicine injections including Xiyanping injection, Xuebijing injection, Reduning injection, Tanreqing injection, Xingnaojing injection, Shenfu injection, and Shengmai injection can be used to treat severe patients with COVID-19, and some clinical research reported the efficiency, there is no systematic review and meta-analyses to investigate the effects and safety of Chinese traditional medicine injections. As far as the current registration situation is concerned, many clinical RCT trials are about to be carried out. Considering this, the present study aims to completely review the effects and safety, which will be of great significance to the clinical treatment of COVID-19.

## Author contributions

**COVID-19 related expertise is provided:** Yanqing Li, Lin Zhang, Hongqiu Zhu

**Data management:** Yulin Li, Haonan Xu, Hui Lang, Lin Bi

**Draft writing:** Yulin Li, Liang Dong, Haonan Xu, Hui Lang

**Manuscript modification and editing:** Yulin Li, Yanqing Li, Haonan Xu, Hui Lang, Lin Zhang

**Methodology:** Yulin Li, Jing Li, Liang Dong

**Program management:** Yulin Li, Haonan Xu, Hui Lang, Lin Bi

**Research design and concept:** Yulin Li, Jing Li, Hongqiu Zhu

**Resource:** Hongqiu Zhu

**Review the manuscript and approve the release:** Yulin Li

**Software:** Yulin Li, Haonan Xu, Hui Lang
